# The economic impact and cost-effectiveness of combined vector-control and dengue vaccination strategies in Thailand: results from a dynamic transmission model

**DOI:** 10.1371/journal.pntd.0008805

**Published:** 2020-10-23

**Authors:** Gerhart Knerer, Christine S. M. Currie, Sally C. Brailsford

**Affiliations:** 1 Mathematical Sciences, University of Southampton, Highfield, Southampton, United Kingdom; 2 Southampton Business School, University of Southampton, Highfield, Southampton, United Kingdom; Brandeis University, UNITED STATES

## Abstract

**Background and aims:**

Dengue fever is a major public health problem in tropical/subtropical regions. Prior economic analyses have predominantly evaluated either vaccination or vector-control programmes in isolation and do not really consider the incremental benefits and cost-effectiveness of mixed strategies and combination control. We estimated the cost-effectiveness of single and combined approaches in Thailand.

**Methods:**

The impacts of different control interventions were analysed using a previously published mathematical model of dengue epidemiology and control incorporating seasonality, age structure, consecutive infection, cross protection, immune enhancement and combined vector-host transmission. An economic model was applied to simulation results to estimate the cost-effectiveness of 4 interventions and their various combinations (6 strategies): i) routine vaccination of 1-year olds; ii) chemical vector control strategies targeting adult and larval stages separately; iii) environmental management/ public health education and awareness [EM/ PHEA]). Payer and societal perspectives were considered. The health burden of dengue fever was assessed using disability-adjusted life-years (DALYs) lost. Costs and effects were assessed for 10 years. Costs were discounted at 3% annually and updated to 2013 United States Dollars. Incremental cost-effectiveness analysis was carried out after strategies were rank-ordered by cost, with results presented in a table of incremental analysis. Sensitivity and scenario analyses were undertaken; and the impact and cost-effectiveness of *Wolbachia* was evaluated in exploratory scenario analyses.

**Results:**

From the payer and societal perspectives, 2 combination strategies were considered optimal, as all other control strategies were dominated. Vaccination plus adulticide plus EM/ PHEA was deemed cost-effective according to multiple cost-effectiveness criteria. From the societal perspective, incremental differences vs. adulticide and EM/ PHEA resulted in costs of $157.6 million and DALYs lost of 12,599, giving an expected ICER of $12,508 per DALY averted. Exploratory scenario analyses showed *Wolbachia* to be highly cost-effective ($343 per DALY averted) vs. other single control measures.

**Conclusions:**

Our model shows that individual interventions can be cost-effective, but that important epidemiological reductions and economic impacts are demonstrated when interventions are combined as part of an integrated approach to combating dengue fever. Exploratory scenario analyses demonstrated the potential epidemiological and cost-effective impact of *Wolbachia* when deployed at scale on a nationwide basis. Our findings were robust in the face of sensitivity analyses.

## Author summary

Dengue fever has become a major public health problem. It is considered one of the most important mosquito-borne viral diseases and occurs in >100 countries in tropical and subtropical regions of Asia-Pacific, the Americas, the Middle East, and Africa with >3 billion people at risk. Despite current control interventions against dengue fever in endemic countries, the disease is associated with considerable healthcare utilisation, personal costs to patients and caregivers, productivity loss and human suffering. Whilst the illness is well understood, there is also recognition that current control efforts focussing predominantly on *Aedes aegypti* control and elimination are less than optimal although may still have an important role to play in the short to medium term. In this study, we consider the cost-effectiveness of individual as well as mixed dengue control strategies in Thailand, embracing chemical interventions, public health education/ environmental control and paediatric vaccination using a dengue vaccine profile broadly consistent with (dengue) vaccines in late stage development. To anticipate the transition to possible new vector control technologies, we also carry out exploratory scenario analyses of the impact and cost-effectiveness of the release of *Wolbachia*-infected mosquitoes (which are less capable of spreading viruses). Our findings indicate that single dengue control interventions can be cost-effective weapons in reducing dengue infections, although their effectiveness may be additionally enhanced when combined.

## Introduction

Dengue fever is a mosquito-borne disease caused by serologically related, but distinct, viruses grouped into four serotypes (DENV-1 to DENV-4). The disease is an important public health problem in more than 100 countries in tropical and sub-tropical regions of Asia-Pacific, the Americas, the Middle East, and Africa with a considerable burden in terms of disease incidence, cost and impact on quality of life. *Aedes aegypti* mosquitoes are the primary vector of transmission for dengue fever and, to a lesser extent, *Aedes albopictus*. These mosquitoes are also responsible for the transmission of other vector-borne diseases including zika virus, chikungunya and yellow fever. It is estimated that 3–4 billion people are at risk of dengue with approximately 390 million dengue infections (95% credible interval: 284–528 million) occurring every year, of which 96 million (95% credible interval: 67–136 million) are symptomatic [1]. The reasons for the growth in dengue fever and severe dengue as leading public health challenges tend to be multi-factorial [2]. These include rapid population growth, increasing unplanned urbanisation, overseas air travel and deteriorations in public health infrastructure [3–5].

When assessing the economic impact associated with dengue, estimates of the annual cost of illness range from approximately $1 billion [6] at the regional level to approximately US$8.9 billion (95% uncertainty interval $3.7–19.7 billion) globally in 2013, with 18% of cases being admitted to hospital and the remaining 48% and 34% of cases classified as ambulatory and non-medical, respectively [7]. Research suggests that almost US$1 billion [6] was spent each year in South-East Asia to treat dengue illness during 2000–2010, with Indonesia and Thailand responsible for 34% and 31% of the total, respectively. Approximately US$451 million of these costs were direct costs [6]. More recent figures using dengue incidence estimates from the Global Burden of Disease Study 2013 [8] suggest that the aggregate cost of dengue in the Southeast Asia, East Asia, and Oceania super-region was approximately $4.8 billion (95% uncertainty interval $1.9–10.8 billion) [7]. Analogously, it has been estimated that the corresponding cost of dengue in the Latin America and Caribbean super-region was approximately US$1.73 billion (95% uncertainty interval $0.72–3.90 billion) in 2013, with Brazil accounting for more than 40% of the total economic burden of dengue in the region [7]. In addition to costs specific to dengue disease, the reported costs of routine (dengue) vector control programmes range from approximately US$0.20 to $38 per inhabitant per year; the median cost being around US$2.50 per inhabitant per year [9].

Estimates of the burden of dengue as part of the Global Burden of Disease 2013 study indicated that the disease was responsible for approximately 1.14 million disability-adjusted life years (DALYs) globally [8,10]. This figure subsequently increased to 2.92 million DALYs globally in a 2017 update [10]. Regional estimates from the 2013 study [8] suggested that approximately 596,700 DALYs and 74,100 DALYs in South-East Asia and Latin America, respectively, were attributable to dengue fever, with the former having the highest rate of DALYs lost due to dengue illness followed by Latin America. The disparity in numbers between the two regions may be partially explained by the higher incidence rates of severe dengue (i.e. dengue haemorrhagic fever [DHF] and dengue shock syndrome [DSS]) in South-East Asia compared to the Americas, as well as the higher case fatality rate [11] in the former. DALY estimates specific to Thailand range from 427 to 471 DALYs per million population [8,12,13].

Other important elements should also be considered in order to estimate the broader economic burden of dengue; for example, possible detrimental effects on foreign direct investment resulting from the incidence of disease and dengue in particular [14–17], as well as the potential impact of dengue disease on tourism revenues [18–20].

At present, the widespread prevention and control of dengue fever is limited to the avoidance of mosquito bites, vector control measures and community engagement for environmental management initiatives [21]. Treatment is made up of supportive care, in the absence of licensed anti-viral prophylactic and/ or therapeutic therapies. Thailand’s dengue control strategy is derived from WHO guidelines [21], which consist of 3 key elements: 1) avoiding transmission by preventing mosquito bites in infected dengue patients; 2) active community detection of non-consulting cases; and 3) vector control strategies comprising environmental management, source reduction, and chemical interventions (adulticide and/ or larvicide) [22]. Carbamates, pyrethroids, organochlorides and organophosphates form some of the most common agents used in insecticide mosquito control, primarily by treatment of water storage containers through larviciding and/ or perio-domestic space spraying, with the mechanism of action targeting the nervous system of the mosquito [21,23,24]. Anecdotal evidence suggests that local vector control ‘at best’ delays infection and has only a marginal impact on the total burden of disease, whilst large-scale control can have a considerable impact [25]. Oft cited and successful examples of systematic vector control campaigns include breeding site elimination during the construction of the Panama Canal and *Aedes aegypti* eradication in Central and Latin America during the 1950s and 1960s [26]. It has been asserted that if appropriately carried out, the suppression of *Aedes aegypti* (i.e. reduced to levels below which epidemics cannot be sustained) can be a pragmatic way to control urban dengue, yellow fever and chikungunya [27]. In addition to the more traditional methods of vector control referred to above, innovative ‘technologies’ are also undergoing evaluation, including *Wolbachia* infection, in which mosquitoes that carry *Wolbachia* bacteria (which is harmless to humans) are released. These mosquitoes and their offspring are less able to carry and spread viruses as the *Wolbachia* bacteria compete with the viruses. There is growing evidence of the effectiveness of large-scale deployments of *Wolbachia-*infected mosquitoes across different geographies, including Yogyakarta, Indonesia (76% reduction in dengue transmission [28]), Niteroi, Brazil (73% reduction in notified dengue incidence [28]), Nha Trang, Vietnam (86% reduction in dengue incidence [28]) and Kuala Lumpur, Malaysia (40% reduction in dengue incidence [29]). Notwithstanding this, commentators have suggested that current (insecticide-based) approaches will likely play a continuing role in vector control frameworks for the foreseeable future, given the relatively long lead time required for widespread implementation of new control measures [30]. Current guidance from the World Health Organization (WHO) ‘…‥encourages affected countries [in relation to both dengue and zika viruses] and their partners to boost the use of current mosquito control interventions as the most immediate line of defence, and to judiciously test the new approaches that could be applied in future’ [31]. Undoubtedly, effective (and widespread) vector control has been problematic to achieve due to resourcing constraints (outside of outbreaks), poor planning, high costs and a lack of a community engagement and acceptance to name but a few [27]. Insecticide resistance is also a growing problem [32]. In this regard, data on mechanisms and prevalence of resistance at specific geographic locations is relatively scarce, although such knowledge may be pertinent to guide national vector control programmes as to the most effective agents to employ in each resistance setting [30].

With respect to dengue control by means of vaccination, the WHO has indicated that ideally, a dengue vaccine should be given in the form of a single dose, protect against all four (dengue) virus serotypes, provide long-term immunity and cause no serious adverse effects [33]. At present, only one dengue vaccine has been licensed although uptake has been relatively low [34]. A number of other dengue vaccines are also in development, including one that has Phase 3 overall dengue vaccine efficacy results [35,36]. Real-world regulatory experiences to date attest to the critical and almost unique complexities that (dengue) vaccine manufacturers face in relation, but not limited, to differential impacts on dengue ‘sero-negatives’ vs. ‘sero-positives’ as well as varying vaccine efficacy against dengue serotypes.

In examining the cost-effectiveness of dengue control strategies, evaluations to date have tended to consider vaccination [37–48] or vector-control programmes [49–56] singularly and not really considered the costs and benefits of mixed strategies as part of combination control. This state of affairs is changing to some extent with recent papers examining, for example, the epidemiological impact of vector control methods in Brazil [57], mathematical modelling of dengue spread with different interventions, including vaccination, larvicide, insecticide and mechanical control [58] and a recent economic evaluation of vector control in the context of a licensed dengue vaccine in different countries [59]. Notwithstanding the evident merits of vaccination in general, arguments exist for the continued importance of vector control in the management of dengue fever [56,60,61]. Its dependence on the *Aedes aegypti* mosquito for transmission [31] means that vector control strategies are likely to also reduce the incidence of the zika virus, yellow fever and chikungunya. Accordingly, vector control tools can play a wider role in controlling and eliminating vector-borne diseases other than dengue. Indeed, one commentator appropriately captured this sentiment: ‘…even if commercial dengue vaccines are available soon after a successful licensure process, vector control is critical to disrupting the epidemiologic triad of dengue and other emergent/resurgent mosquito-borne viruses that *Aedes aegypti* can also transmit. Thus, an integrated approach focusing on the mosquito vector cannot be disputed’ [24].

If we accept the premise that an easy solution for dengue control does not exist and that multiple strategies are likely be more effective than a stand-alone strategy, the pertinent question then becomes what the cost-effective options are from a priority setting and decision-making perspective. In this regard, we consider a number of dengue control options, both individually as well as in combination, encompassing historical forms of vector control as well as possible new ones in the form of vaccination and *Wolbachia*, to determine the best intervention(s) for controlling the spread of dengue from a cost-effectiveness perspective. We treat orthodox vector control as the foundation of dengue prevention before introducing vaccination over time in the form of a staggered (national) ramp-up to examine the costs and effects of different combined control strategies. We then anticipate the possible transition to a new control context in the form of *Wolbachia* in subsequent exploratory scenario analyses.

We carried out exhaustive experiments to determine the impact of varying factors including the costs of interventions, vaccination coverage, intensity of vector control, disutility weights and discount rates amongst others. In the next section, the model is briefly described, followed by a presentation of results, discussion and ending with conclusions and next steps.

## Methods

We assessed the impact of different control interventions in Thailand using our previously published dengue dynamic transmission model [62] and incorporating updated data inputs and interventions. The model provided the epidemiological base for economic analyses, where we assumed an epidemiology that was representative of average Thailand dengue epidemiology in the years 2008–2012, linking dengue incidence to costs and outcomes, and predicted the number of dengue cases at steady state and under each control strategy. This was subsequently combined with economic inputs to report the costs and consequences of different strategies and included formal cost-effectiveness analysis. As vaccination is a continuous intervention, its effects accumulate over the years that follow introduction. Conversely, environmental management/ public health education and awareness (EM/ PHEA), larvicide or adulticide tend to be relatively short-term interventions, therefore their effects are evident much sooner. To ensure equivalence of comparison, the impact of all interventions in the form of cumulative costs and consequences were estimated over 10 years following intervention initiation. This follow-up period was considered to correspond to reasonable timescales for public health decision-makers [63,64]. In exploratory scenario analyses, we considered the impact of *Wolbachia* individually and in combination with vaccination and took time frames longer than 10 years into account.

### Epidemiology model structure

In brief, the transmission model simulated the population-wide transmission dynamics of symptomatic dengue fever in Thailand, focusing on consecutive dengue infections and the overall impact of control interventions on dengue incidence. The model assumed that the four dengue serotypes have comparable infectiousness and prevalence (as a simple proxy for complex dengue virus circulation dynamics) as opposed to modelling the behaviour of individual dengue serotypes. This is consistent with other modelling studies in the field [65–68]. The model incorporated the age structure of the population, cross-protection, combined vector-host transmission and a climatic factor simulating seasonal influences in the mosquito population. Further information including the flow diagram of the infection process is provided in S1 Appendix (Methods–Additional Details).

### Data

The model [62] was calibrated using dengue national surveillance data [69] stratified by type of management (inpatient vs. outpatient), and age group (0–4, 5–9, 10–14, 15–24, ≥25 years) assuming steady state and adjustment for under-reporting [70]. Further details concerning data, expansion factors and calibration are contained in S1 Appendix (Methods–Additional Details).

### Interventions

Health and economic outcomes were evaluated for combinations of the following interventions.

#### Chemical control (insecticide and larvicide applications)

The evidence base as to the effectiveness of vector control is somewhat undeveloped with a relative deficit of randomised controlled trials measuring epidemiological (as opposed to entomological) impact. For example, a cluster randomised trial evaluating community mobilisation for dengue prevention showed a lower risk of infection with dengue virus in children (relative risk reduction 29.5% [95% confidence interval: 3.8%–55.3%]) and lower reports of dengue illness (relative risk reduction 24.7% [95% confidence interval: 1.8%–51.2%]) [71]. A meta-review [72]–comprised of thirteen systematic reviews investigating the effectiveness of *Aedes* control interventions or protective measures against *Aedes*-transmitted diseases–determined the strength of the evidence to be consistently low or very low and recommended that future evaluative research efforts employ a randomized controlled trial paradigm with longer durations of follow-up and accompanying disease-related metrics. Specifically, a systematic review of the effectiveness of periodomestic space spraying (pyrethroids, pyrethrins or organophosphates) demonstrated reductions in different entomological measures, but the effects disappeared within days or weeks [73]. The authors concluded that more research was needed. Similarly, a systematic literature review of the effectiveness of a commonly used larvicide (temephos) found that larvae were controlled for approximately 2–3 months in the context of a single community-based intervention dependent on an array of factors including study design, local circumstances, water turnover rates and season [74].

Consistent with other authors (e.g. Burattini et al. [75], Luz et al. [54,76] and Fitzpatrick et al. [59]), we modelled the effect of vector control as a reduction in the vector population (as the means of dengue transmission). In this regard, the impact of chemical larvicide and adulticide interventions was simulated by increasing mortality rates for both aquatic life forms (egg, larval and pupal stages) and adult mosquitoes using the square pulse function in Berkeley Madonna [77]. The incidence of insecticide has increased greatly in recent years [30] and evidence indicates that continuous use of chemical control increases the potential for insecticide resistance with clear negative externalities for other animal species, as well as the natural environment due to toxicity of such compounds [24]. Moreover, mathematical modelling simulations suggest that increased applications of insecticide lead to decreasing reductions in mosquito abundance with a tipping point identified in the frequency of insecticide applications after which there are diminishing returns [54,76]. Luz et al. additionally report that continuous chemical-based vector control may subsequently worsen epidemics due to the evolution of insecticide resistance [54,76]. Analogously, and in reference to sterile insect release techniques (SIT), White et al. [78] state that “….models that assume a constant release strategy will tend to over-estimate the true level of population control”. We did not include SIT interventions in the present study but suggest that such over-estimation (referred to above) is as relevant for chemical interventions as for genetically modified insect interventions. Accordingly, in the current study, chemical control was modelled as discrete periodic interventions, rather than continuous, targeted 1 day per week over 3 weeks at the beginning of the annual dengue season. We used mortality rates of 30% to simulate low-efficacy adulticide and larvicide consistent with the low-efficacy chemicals frequently used in real-world conditions [75,79,80]. We evaluated the impact of 3 applications of larvicide/ adulticide (i.e. separate applications 1 day per week for 3 weeks each year over 5 years) as part of combined dengue control strategies. This was informed by both empirical field trials, which found that approximately 2–4 insecticide applications annually were optimal [81] and the results of mathematical modelling, which suggested that combined vector control was superior to single interventions [54,76].

#### Environmental management/ public health education & awareness

Embracing mechanical control, breeding site/ source reduction and associated educational campaigns (focused on training/ awareness of the local populace with the aim of reduction/ elimination of breeding sites–‘clean-up’ campaigns). Whilst such initiatives are rarely used as the sole control measure, they are nevertheless considered essential to reducing breeding sites and disrupting disease transmission [72].

Notwithstanding this, the evidence base is relatively scarce, although there is a meta-review which included 4 reviews (5 study arms) that reported on educational and awareness campaigns [72]. Only 1 of the studies/ study arms reported dengue incidence as the main outcome measure and was considered low quality. The remainder reported entomological indices as the main outcomes measures and were deemed very low quality [72]. A more recent systematic review and meta-analysis for the effectiveness of environmental dengue vector control methods [82] focused on (i) container covers with and without insecticides; (ii) waste management and clean-up campaigns and (iii) elimination of breeding sites by removal and/ or making unusable potential mosquito breeding sites. The authors indicated that the great majority, if not all, of the dengue vector control interventions under study showed some form of effectiveness in reducing larval/ pupal densities of *Aedes* mosquitoes, although they strongly advocated for additional and comparable high-quality studies to strengthen the evidence base, ongoing engagement of communities and public health experts and information on cost-effectiveness and long-term sustainability [82].

In terms of real-world observation, results from a recently published mosquito control programme in Sri Lanka attest to the importance of such interventions and indicate that approximately 2,200 cases of dengue were averted during the 31 months of the intervention, resulting in a 57% reduction in dengue incidence [56]. The programme aimed to reduce mosquitoes in high-risk hotspots with large-scale systematic ‘door-to-door’ inspections. Mixed teams comprising public health officials, police and military personnel carried out daily inspections in numerous locations to identify and remove typical mosquito breeding sites, such as containers of stagnant water. The programme supplemented routine mosquito control interventions with insecticides and larvicides.

In the present study, EM/ PHEA was represented in model simulations by reductions in carrying capacity (K), the assumption being that reducing environments favourable to the breeding of *Aedes aegypti* vectors reduces the population. Previous simulations of the impact of breeding source reduction on vector-borne disease have used 40–70% reductions in carrying capacity [62,79,83]. For example, in simulations of the impact of analogous control on the burden of chikungunya, Dumont and Chiroleu [79] showed that the best results were obtained with a 66% reduction in carrying capacity. However, they felt that this figure was unrealistic and a decrease of 25% was more plausible under real-world conditions [79]. Consequently, we used the more conservative figure of a 25% decline in carrying capacity to simulate the impact of EM/ PHEA. Reflecting the ongoing nature of this package of interventions, the aquatic carrying capacity was reduced for the duration of the dengue season and beyond, approximately days 100–250 in the calendar year equating to higher temperatures and rainfall. This was done for 1 year only and the effects were evaluated over 10 years [79,83].

#### Vaccination

This acts on susceptible persons with the numbers governed by the balance between vaccine efficacy, vaccination coverage and waning of protection. We adopted a dengue vaccine profile approximately consistent with (dengue) vaccines in late stage development and applied certain assumptions in this regard. Namely, that the vaccine has an overall protective efficacy of 80% (falling to 73% at 18 months post-vaccination and assumed constant at this level until the end of study follow-up) in all populations and against all grades of dengue fever (i.e. vaccine efficacy is not a function of age or severity) and with a duration of protection of 10 years. Additionally, it was assumed that the vaccine is effective after a course of vaccination, does not distinguish between seronegatives and seropositives (i.e. protects both) and has no adverse events nor serious adverse events (breakthrough infections). Consistent with analyses undertaken in our previous publication [62], we assumed that dengue vaccination would form part of routine paediatric vaccination and fit into existing child immunisation schedules at 1 year and under (in the model, vaccination was administered at 12 months of age). In the base case, we applied vaccination coverage of 80% with roll-out staggered over 4 years, i.e. 20% coverage in the first year, 40% coverage in the second year, 60% coverage in the third year and 80% coverage at the beginning of the fourth year post roll-out. When considering vaccination in combination with *Wolbachia* as part of the exploratory scenario analyses, it was assumed that vaccination coverage had arrived at steady state with no delay in implementation, i.e. there was no ramp-up period. We also examined different population vaccination coverages of 40% and 60%.

#### Wolbachia

This is a potential intervention for arbovirus control that has demonstrated the ability to circulate amongst wild *Aedes aegypti* populations in field trials [84,85]. Whilst primarily intended as a means to control dengue virus transmission, it also has applications to chikungunya and zika virus, which share the same vector of transmission [86]. Potential outcomes of wild-type mosquitoes being infected with *Wolbachia* may include reduced egg-laying rates, reduced mosquito population, shorter (mosquito) lifespan and reduced transmission capabilities, which can greatly decrease the potential to spread mosquito-borne viral diseases (such as referred to above). A *Wolbachia* replacement strategy and mechanism of action involves the release of *Wolbachia*-infected mosquitoes into the natural mosquito environment, which subsequently mix and breed with the native wild mosquitoes. *Wolbachia* infection takes place during reproduction resulting in the transformation of wild-type mosquito environments into *Wolbachia-*infected environments as the process replicates itself over generations of mosquitoes. Researchers have captured relevant differences between mosquitoes (*Wolbachia*-infected/ non-*Wolbachia* infected) both explicitly (i.e. modelling *Wolbachia-*infected mosquitoes) and/ or implicitly (i.e. focusing on parameters affected by *Wolbachia*) in assorted models of differing complexity (e.g. Dorigatti et al. [87], Ndii et al. [88], Xue et al. [89], Shen [90], Bañuelos et al. [91], O’Reilly et al. [92]). The authors variously employed scaling factors to reflect the evidence of, for example, changes in birth/ reproduction/ maturation (from aquatic to adult mosquito stage) rates, mortality and biting rates and human vector transmissibility [88,89] due to *Wolbachia* infection. In this regard, mortality rates of *Wolbachia*-infected mosquitoes (wMel strain) are higher than non-*Wolbachia* vectors (scaling factor >1 × μv) as evidence shows that *Wolbachia* infection reduces the mosquito lifespan [87–90]. Similarly, *Wolbachia* infection is thought to hinder mosquito feeding and decrease the (successful) biting rate (scaling factor <1 × b) [88,89] due to a condition known as bendy proboscis. In turn, a reduced biting rate also means that the overall human-to-vector transmission rate is reduced as some *Wolbachia*-infected mosquitoes may not be infected with dengue virus due to a process known as ‘viral replication inhibition’ (scaling factor <1 × βv) [88,89,91].

In exploratory scenario analyses, we considered the predicted impact and cost-effectiveness of a country wide *Wolbachia* programme (wMel strain), singularly and in combination with vaccination. Given the exploratory nature of these analyses, we made a number of simplifying assumptions and compared long-term epidemiological projections with previous authors [87] as a basic validation check. We focused only on the situation where *Wolbachia*-infected mosquitoes arrive to steady-state/ fixation in the (mosquito) population after a period of release and the possibility to reduce or eliminate the disease in the human population. Therefore, we were not interested in such factors as the necessary and sufficient conditions for *Wolbachia* penetration and propagation in the *Aedes aegypti* population nor the optimal release strategy. For example, we did not model *Wolbachia-*infected mosquitoes explicitly, rather, model parameters impacted by *Wolbachia*, including mosquito death and biting rates and transmissibility of infection were modified (using scaling factor estimates derived from the literature), to convert non-*Wolbachia* parameters to *Wolbachia*-infected parameters [88,89]. The scaling factors used in our analyses are presented in Table 1.

**Table 1:** Scaling factors to convert non-*Wolbachia* vector parameters to *Wolbachia*-infected vector parameters.

*Wolbachia* Strain	Decreased birth/ reproductive/ maturation rate	Increased mortality rate	Decreased biting rate	Decreased transmission rate
*Wolbachia* free	1.00	1.00	1.00	1.00
wMel	0.95	1.10	0.95	0.50

#### Combination interventions

Descriptions of the 5 combination dengue control strategies are presented in Table 2.

**Table 2:** Combined dengue control strategies–glossary.

Strategy	Combination Dengue Control
**A**	No intervention (steady state)^a^
**B**	Adulticide^b^ (3 applications); larvicide^b^ (3 applications)
**C**	Adulticide^b^ (3 applications); EM/ PHEA^c^
**D**	Adulticide^b^ (3 applications); larvicide^b^ (3 applications); EM/ PHEA^c^
**E**	High-coverage (80%) vaccination; adulticide^b^ (3 applications); EM/ PHEA^c^
**F**	High-coverage (80%) vaccination; adulticide^b^ (3 applications); larvicide^b^ (3 applications); EM/ PHEA^c^

^a^ Number of infections at steady state in Thailand, all ages combined

^b^ Discrete applications of limited duration (1 day) at start of and/ or during dengue season over 5 years; 3 applications per dengue season for 5 years

^c^ 25% reduction in carrying capacity, *K*, of immature stages over 1 year

EM/ PHEA = environmental management/ public health education and awareness

### Dengue severity

The presence and severity of symptoms determine the associated costs and impact on quality of life. The severity of infection was not introduced directly into the epidemiological transmission model; rather the transmission model generated the overall number of infections and the probability of different manifestations of dengue (symptomatic–dengue fever/ severe–DHF and DSS) were subsequently applied in the economic model to derive costs of dengue by severity.

### Outcomes

The humanistic burden of dengue fever was assessed by calculating DALYs lost to disease using the methodology described by Murray and Lopez [93,94]. The duration of symptoms was different for symptomatic (dengue fever) and severe (DHF/ DSS) disease to take into account the difference in their impact on quality of life.

To enable comparison with DALYs lost to dengue presented in other studies [12,40,95,96], we applied comparable values for discounting functions (C, b and r) derived from the Global Burden of Disease study [94]. We did not consider age weighting in the base case but examined the impact of this in sensitivity analyses. Disability weights, D, were obtained from Carrasco et al. [40], Durham et al. [41] and Lee et al. [39]; these studies also considered the cost effectiveness of a potential dengue vaccine.

We adopted the approach of Clark et al. [12] and assumed that unreported cases are likely less severe than reported cases, although they may still hinder usual daily activities, but for a shorter length of time. Consistent with this, we assigned similar disability weights to unreported cases as to reported cases of dengue fever, but for a shorter duration (4 days for unreported; 10 days for reported).

Inputs used to calculate DALYS lost are presented in S1 Appendix Table 1.

### Perspective

Both payer and societal perspectives were considered.

### Costs

We used cost estimates of a dengue fever episode obtained from Shepard et al. [6]. These values were based on a study by Kongsin et al. [97], which used the same cost data as Suaya et al. [98]. Kongsin et al. [97] assessed the costs of dengue fever to Thai society and included direct medical costs incurred within the government public health system and borne by patients and households, direct non-medical costs and productivity loss (i.e. indirect costs to households for loss of income and absence from school including caregiver and patient days lost other than for school or work).

Additional studies with applicable unit costs [13,99] that have been used by other researchers–for example, Lee et al. [39]–were not considered in the present study due to their reliance on expert opinion, secondary data or being considered somewhat outdated, leading to potential under-estimation of costs [6]. Accordingly, unit costs (per dengue fever episode) derived from Shepard et al. [6] were used to estimate the following costs:

- Payer perspective:
  ◦ direct medical costs for inpatient and outpatient dengue cases
- Societal perspective:
  ◦ direct medical costs for inpatient and outpatient dengue cases
  ◦ direct non-medical costs for inpatient and outpatient dengue cases
  ◦ indirect costs for inpatient and outpatient dengue cases.

Total costs were comprised of direct medical costs and intervention costs (detailed below) from the payer perspective and direct medical costs, direct non-medical costs and indirect costs in addition to intervention costs from the societal perspective.

As part of sensitivity and scenario analyses, we substituted unit costs with other sets of unit costs referred to above, as well as others. For example, healthcare unit costs (excluding vaccine costs and/ or vector control costs) reported in Lee et al. [100], Fitzpatrick et al. [59] and Flasche et al. [42]. Cost inputs are presented in Table 3.

**Table 3:** Input values.

Input	Base Case	Sensitivity Analysis
**Duration of vaccine protection**	10 years	5 years and lifetime perspective of the first vaccinated cohort
**Vaccine efficacy**	80% (falling to 73% at 18 months)	73–85% (falling to 67–79% at 18 months)
**Vaccine coverage**	- Routine vaccination at 1 year of age (80% coverage)	- Routine vaccination at 1 year of age (40% coverage) - Catch-up vaccination for those children aged <1 year and <5 years; different levels of catch-up vaccination coverage scenarios: ◾ moderate (50%) ◾ low (30%)
**Discount rates**	3% for costs and effects	3% for costs, 1.5% for effects; undiscounted results
**Time horizon**	10 years	5 years
**DALY utility weights, D**	0.211 and 0.5 for symptomatic cases of DF and DHF/ DSS, respectively [40]	- 0.197 and 0.545 for symptomatic cases of DF and DHF/ DSS, respectively [39,41] - 0.37 and 0.52 (children) and 0.42 and 0.53 (adults) for symptomatic cases of DF and DHF/ DSS, respectively^a^ [40]
**DALY age-weighting parameter, *β***	No age weights	Age weights
**Vaccine price per course**	$40 plus $4 vaccine administration costs	$20 and $60 plus $4 vaccine administration costs
**Cost of ‘un-reported’ cases**	$12.12 for clinic visit [39]	$0 and $40 for clinic visit [42]
**Inpatient costs**	- $266 DF inpatient direct medical costs [98] - $566.43 DHF inpatient direct medical costs [6] - $72.77 inpatient direct non-medical costs [6] - $54.59 inpatient indirect costs [6]	- Unit cost profiles from Fitzpatrick et al. [59], Lee et al. [100] and Flasche et al. [42] in scenario analyses
**Outpatient costs**	- $141.61 outpatient direct medical costs [6] - $82.20 outpatient direct non-medical costs [6] - $13.65 outpatient indirect costs [6]	- Unit cost profile from Fitzpatrick et al. [59], Lee et al. [100] and Flasche et al. [42] in scenario analyses
**Number of vector control interventions**	- 3 (1 day per week over 3 weeks at beginning of annual dengue season)	- 2 (1 day per week over 2 weeks at the beginning of annual dengue season)
**Vector control unit costs**	- $354,098 for 3 applications of larvicide or adulticide per million persons per annum [54] - $382,482 for EM/ PHEA programmes per 1 million persons [101,102]	- $277,724 for 2 applications of larvicide or adulticide per million persons per annum [54]

^a^ Values are mean disability weights for symptomatic ambulatory and hospitalised children and adults but were applied in this study as a proxy for disability weights of symptomatic cases of DF and DHF/ DSS

DALY = disability-adjusted life-year; DF = dengue fever; DHF = dengue haemorrhagic fever; DSS = dengue shock syndrome; EM/ PHEA = environmental management/ public health education and awareness

#### Costs of unreported cases

Where costs were ascribed to unreported cases for type of treatment, it was assumed that these were on an outpatient basis only in line with the likely less severe nature of these cases [12]. Unreported hospitalisations and deaths have been documented and some estimations for hospitalisations exist for Thailand [6]. Notwithstanding this, we employed a conservative approach in the estimation of these costs. Consequently, we assumed that there were no hospitalisations or deaths associated with unreported cases.

#### Intervention costs

For chemical vector control, we employed a similar cost structure to Luz et al. [54], who assumed annual costs of $201,350 and $277,724 per million persons for 1 and 2 applications of larvicide or adulticide, respectively, inflated to USD 2013. The authors assumed that the cost of a third application of larvicide or adulticide per million population was the same as the incremental cost of going from one to two applications (i.e. an additional $76,374) [54] for a total cost of $354,098 (USD 2013) for 3 applications of larvicide or adulticide per million population. To derive the costs of a Thai-wide vector control programme comprising 3 applications of larvicide or adulticide, the latter cost was then multiplied by the Thai population index. This equates to approximately $0.0295 per capita per month ($0.354 per capita per annum) and compares to other vector control estimates documented in the literature, for example, Undurraga et al. [103] and Fitzpatrick et al. [59]. The latter presented estimates of sustained vector control for Thailand of 2013 USD 0.055 (range 0.033–0.088) per capita per month ($0.66 per capita per annum).

For the costs of environmental management–embracing source reduction, sanitation improvements and health education and awareness measures–we derived cost estimates from Packierisamy et al. [101,102]. They collected information on capital and recurrent expenditure for dengue vector control activities in Malaysia. Data were recorded by line item and function; line items consisted of personnel, administrative and storage buildings, vehicles, fumigation equipment, pesticides, personal protective equipment and out-sourcing of fumigation services to private companies. Functions included a breakdown of costs by inspection, entomological surveillance, fumigation, larviciding and health education. We used the per capita costs of health education ($0.35) to derive the costs of environmental management (embracing source reduction, sanitation improvements and health education measures) per million persons ($350,000). Cost estimates were then updated to USD 2013.

For vaccination, we use a cost of $40 per vaccination course and assumed vaccine administration costs of $4.

Due to uncertainty in the costs of a *Wolbachia* intervention and in line with the exploratory nature of these analyses, we used 2 different cost estimates to evaluate the potential cost-effectiveness of *Wolbachia*: firstly, a cost per dengue case averted of $1 (which we then used to back-calculate a cost per person of $4.45) and secondly, a cost per person of $1 (the latter being an aspirational cost of the World Mosquito Programme *Wolbachia* method). These costs were assigned over 4 years to simulate accelerated *Wolbachia* implementation to the point where *Wolbachia*-infected mosquitoes have reached steady state/ fixation in the population.

#### Productivity costs due to death

The economic costs of premature mortality (in terms of productivity loss and lifetime earnings foregone) were not included in the cost-effectiveness analyses due to concerns over the risk of double counting benefits associated with averted deaths [104,105].

### Discount rate

Costs were discounted at 3% per annum as suggested by Thailand’s Health Technology Assessment guidance and the WHO [106,107].

### Cost-effectiveness analysis

The cost-effectiveness of different dengue control strategies was evaluated in terms of the incremental cost per DALY averted. In the first instance, dengue control strategies were rank-ordered by increasing cost with all strategies that were both costlier and less effective than alternative strategies (i.e. ‘strongly dominated’) subsequently eliminated. The incremental cost-effectiveness ratio (ICER) was then calculated for the remaining strategies compared to the next least expensive strategy by dividing the additional cost by the additional benefit to derive the incremental cost per DALY averted. Next, all strategies that were ‘weakly dominated’ (i.e. the ICER for this strategy was higher than that of the next more effective alternative) were eliminated and the ICER for the remaining strategies was then re-calculated. Results are presented in the form of a table of incremental analyses, i.e. the set of potentially cost-effective options.

Frequently cited cost-effectiveness thresholds [108–110] relate to a country’s per capita gross domestic product (GDP) suggesting that ‘interventions that avert one DALY for less than average per capita income for a given country or region are considered very cost-effective; interventions that cost less than three times average per capita income per DALY averted are still considered cost-effective; and those that exceed this level are considered not cost-effective [109]’. Whilst not designed to be applied mechanistically, these categories act to provide useful guidance alongside additional contextual information such as affordability, budget impact, fairness, feasibility and other criteria appropriate to the local context [111]. Notwithstanding this, Thailand is one of the few middle-income countries to have a locally established threshold to guide decision-making. In this regard, the threshold criteria for cost-effective health interventions in Thailand was approximately 120,000 Thai Baht [THB] (from 2012 onwards [112], equivalent to $3,860 in 2013 USD with a conversion rate of $1US = 31.0914 THB as of 30 June 2013). This threshold was subsequently increased to the current level of 160,000 THB (equivalent to $5,146 in 2013 USD with a conversion rate of $1US = 31.0914 THB as of 30 June 2013).

### Sensitivity analysis

In this study, probabilistic sensitivity analysis was not carried out for practical reasons related to model run-time and complexity. Rather, the sensitivity of interventions in the table of incremental analyses to changing assumptions was explored by univariate variation of key parameters and then iteratively recalculating incremental analyses for the control strategies under evaluation. Both economic and epidemiological parameters were considered. Sensitivity analysis for epidemiological parameters was restricted to those variables shown to be potentially influential in model analyses reported in previous research. For example, vector mortality, duration of infectious period in host, latent period in vector and biting rate were identified as particularly impactful variables when subject to variation in Bartley et al. [113], from which the current model was adapted. In a similar vein, Amaku et al. [114] found that model parameters related to control (i.e. vector mortality rate, biting rate and immature stage carrying capacity) also proved influential to the relative amount of variation if these parameters were varied by ±1%. Therefore, we examined the relative amount of variation in incremental analyses if epidemiological variables including vector mortality rate, biting rate and carrying capacity of immature stages were modified by ±5%. Duration of infectiousness in host and the latent period in vector were varied by ±1 day. Greater levels of variation in key epidemiological parameters were not possible due to problems in model convergence. The variables under consideration in the epidemiological sensitivity analyses formed part of the calibrated transmission model. However, the model was not re-calibrated after each change in parameter.

Table 3 presents inputs used for the economic model and univariate sensitivity analysis.

## Results

To evaluate the impact of different control interventions, we compared the base-case steady state (without intervention) to the number of dengue cases, outpatient visits, hospitalisations, DALYs lost and deaths over a 10-year period following the introduction of single and combined dengue control interventions before subsequently carrying out cost-effectiveness analyses. Model results are available in S2 Appendix (Dengue CEA Decision Tree Results).

### Outcomes

At steady state, the simulation model predicted approximately 7 million symptomatic dengue infections for all age groups combined in Thailand over a 10-year period adjusting for under-reporting [70,115] (Table 4). Most cases (94%) were attributable to dengue fever, with the balance of cases classified as severe dengue fever cases (combined DHF/ DSS). This translated into approximately 890 dengue-related deaths with a cumulative total of approximately 67,595 DALYs lost over 10 years. For the entire period of follow-up, this equated to an expected dengue burden of 1064 DALYs lost per million persons (average annual dengue burden of 106 DALYs lost per million persons). Additionally, the model predicted approximately 6.5 million outpatient consultations and 625,000 hospitalisations over 10 years.

**Table 4:** Baseline estimates and impact of single vector control interventions and vaccination on dengue burden over a 10-year period (number of cases, outpatient consultations, hospitalisations, deaths and DALYs lost).

Category	No intervention (Steady State)^a^	Adulticide^b^ (× 3)	Larvicide^b^ (× 3)	EM/ PHEA^c^	Vaccination: (80% Coverage)
Total Dengue Cases (millions)	7.147	4.412	6.321	5.462	3.400
Symptomatic	6.684	4.126	5.912	5.108	3.180
Severe	0.463	0.286	0.410	0.354	0.220
Total Outpatient Consultations (millions)	6.523	4.026	5.769	4.985	3.103
Total Hospitalisations (millions)	0.625	0.386	0.552	0.477	0.297
Total Deaths	890	549	787	680	423
Total DALYs lost	67,595	41,731	59,788	51,670	32,132
Total DALYs lost per million population	1064	657	942	814	506

^a^ Number of infections at steady state in Thailand, all ages combined

^b^ Discrete applications of limited duration (1 day) at start of and during dengue season over 5 years; 3 applications per dengue season for 5 years

^c^ 25% reduction in carrying capacity of immature stages, *K* over 1 year

DALY = disability-adjusted life-year; EM/ PHEA = environmental management/ public health education and awareness

### Outcomes–single interventions

Results for single interventions (Table 4) showed that vaccination was projected to result in the lowest burden of disease over 10 years with 32,132 DALYs lost (approximately 506 DALYs lost per million population), representing a 52% reduction from steady state. Of the more orthodox and routine vector control measures, adulticide (administered in 3 discrete applications per dengue season) demonstrated the lowest burden of disease over 10 years (41,731 DALYs lost [–38%] and 657 DALYs lost per million population). The low-efficacy larval control modelled in this study had little impact on the dengue health burden and performed the worst of the single control interventions under this metric.

### Outcomes–combined interventions

Combined control strategies that included vaccination were projected to have the greatest bearing on disease burden, in terms of dengue infections prevented and DALYs lost (Table 5). When considering the impact of combined vector control strategies, these were observed to be largely additive and targeted distinct stages in the vector lifecycle, represented by different entry points in the model (aquatic larvae, adult mosquitoes and carrying capacity). For example, as single interventions, adulticide and EM/ PHEA reduced the disease burden by approximately 38% and 24%, respectively, and in combination, the reduction was approximately 61% (Table 5). However, when vaccination formed part of a mixed control strategy, the combined benefit was less than the sum of the components. For example, vaccination alone led to an approximate 52% reduction in disease burden, but when combined with adulticide (38% reduction alone) and EM/ PHEA (24% reduction alone), only resulted in an overall 79% reduction in disease burden. One potential explanation for this is that routine vector control targeting different channels reduced the force of infection to such an extent that the added impact of vaccination was moderated. This has implications for demonstrating cost-effectiveness as will be seen in the following section. Adding larvicide to this combination resulted in very marginal incremental benefits only.

**Table 5:** Baseline estimates and impact of combined vector control and vaccination interventions on dengue burden over a 10-year period (number of cases, outpatient consultations, hospitalisations, deaths and DALYs lost).

Category	No intervention (Steady state)^a^	A3^b^L3^b^	A3^b^ EM/ PHEA^c^	A3^b^L3^b^ EM/ PHEA^c^	V80A3^b^ EM/ PHEA^c^	V80A3^b^ L3^b^EM/ PHEA^c^
Total Dengue Cases (millions)	7.147	3.618	2.814	2.263	1.483	1.390
Symptomatic	6.684	3.383	2.632	2.116	1.387	1.304
Severe	0.463	0.234	0.182	0.147	0.096	0.090
Total Outpatient Consultations (millions)	6.523	3.302	2.568	2.065	1.354	1.272
Total Hospitalisations (millions)	0.625	0.316	0.246	0.198	0.130	0.122
Total Deaths	890	450	350	282	185	174
Total DALYs lost	67,595	34,223	26,621	21,404	14,022	13,182
Total DALYs lost per million population.	1,064	539	419	337	221	208

^a^ Number of infections at steady state in Thailand, all ages combined

^b^ Discrete applications of limited duration (1 day) at start of and during dengue season over 5 years

^c^ 25% reduction in carrying capacity of immature stages, *K* over 1 year

A3 = adulticide (3 applications); DALY = disability-adjusted life-year; EM/ PHEA = environmental management/ public health education and awareness; L3 = larvicide (3 applications); V80 = vaccination with 80% coverage

### Cost-effectiveness analyses–single interventions

From a societal perspective, total costs for different control programmes, inclusive of intervention costs, ranged from approximately $333 million (EM/ PHEA) to $470 million (larvicide– 3 applications) (Table 6). Larval control exhibited the highest total costs over 10 years, with major cost drivers being associated with the number of severe cases and hospitalisations.

**Table 6:** Cost-effectiveness analysis of single vector-control strategies (societal perspective). ^a^

Strategy	Intervention Costs (millions)	Discounted total		Incremental		ICER $/ DALY Averted^b^
		Intervention + Societal Costs (millions)	DALYs Lost	Intervention + Societal Costs (millions)	DALYs Lost	
EM/ PHEA^c^	$24.288	$332.598	51,670	–	–	–
Adulticide^d^ (× 3)	$106.065	$362.673	41,731	$30.076	9,940	$3,026
No intervention^e^	$0.00	$412.265	67,595	–	–	D
Vaccination (80%)	$227.329	$435.780	32,132	$73.106	9,599	$7,616
Larvicide^d^ (× 3)	$106.065	$470.216	59,788	–	–	D

^a^ All costs were measured in 2013 USD; costs and DALYs were discounted at 3%

^b^ Compared with the preceding non-dominated strategy; small differences due to rounding error

^c^ 25% reduction in carrying capacity of immature stages, *K* over 1 year

^d^ Discrete applications of limited duration (1 day) at start of and/ or during dengue season over 5 years; 3 applications per dengue season for 5 years

^e^ Steady state.

D = dominated; DALY = disability-adjusted life-year; EM/ PHEA = environmental management/ public health education and awareness

EM/ PHEA was the least costly intervention from a societal perspective and therefore formed the reference intervention. Only adulticide (3 applications) and vaccination were not dominated interventions, with ICERs of $3,026 and $7,616 per DALY averted, respectively. When restricting the analysis to the payer perspective (i.e. including only direct medical costs for inpatient and outpatient dengue cases), EM/ PHEA remained the reference intervention as it had the lowest costs (inclusive of intervention costs), with larvicide exhibiting the highest total costs (approximately $403 million). Adulticide and vaccination remained the only non-dominated interventions, with ICERs of $3,986 and $8,540 per DALY averted, respectively.

Therefore, our results indicate that, from both payer and societal perspectives, an adulticide programme made up of 3 discrete applications per dengue season or vaccination (80% coverage) can potentially be considered as cost-effective (if not highly cost-effective) interventions in Thailand according to broader criteria of cost-effectiveness.

### Cost-effectiveness analyses–combined interventions

From the societal perspective, discounted total costs for different combined control strategies ranged from approximately $295 to $554 million over 10 years (Table 7). Adulticide in combination with EM/ PHEA (Strategy C) was the least costly control strategy, whilst Strategy F (vaccination, adulticide, larvicide and EM/ PHEA) was associated with the highest costs but the lowest number of DALYs lost, with the major cost driver being vaccination. Similar to the predicted reductions in disease burden highlighted earlier, decreases in total costs (without vaccination) were observed to be broadly additive in nature. For example, whilst the total costs of EM/ PHEA and adulticide in isolation were $333 million (–19% vs. no intervention) and $363 million (–12% vs. no intervention), respectively, total costs for EM/ PHEA and adulticide in combination were $295 million (–28% vs. no intervention), i.e. less disease burden equates to reduced total costs. Strategies E (vaccination, adulticide and EM/ PHEA) and F (vaccination, adulticide, larvicide and EM/ PHEA) were the only non-dominated strategies with expected ICERs of $12,508 (vs. Strategy C–adulticide and EM/ PHEA) and $120,028 (vs. Strategy E: vaccination, adulticide and EM/ PHEA) per DALY averted, respectively (Table 7). The incremental impact of incorporating larvicide into combined control strategies was not justified by the additional resultant costs. All other combined control interventions were dominated strategies.

**Table 7:** Cost-effectiveness analysis of combined dengue control strategies (societal perspective).^a^

Strategy	Interventions	Intervention Costs (millions)	Discounted Total		Incremental		
			Intervention + Societal Costs (millions)	DALYs Lost (thousands)	Intervention + Societal Costs (millions)	DALYs Lost (thousands)	$/ DALY Averted^b^
**C**	A3^c^EM/ PHEA^d^	$130.353	$295.056	26,621	–	–	–
**D**	A3^c^L3^c^EM/ PHEA^d^	$236.418	$371.576	21,404	–	–	ED
**A**	No intervention^e^	$0.00	$412.265	67,595	–	–	D
**B**	A3^c^L3^c^	$212.130	$424.679	34,223	–	–	D
**E**	V80A3^c^EM/ PHEA^d^	$358.174	$452.639	14,022	$157.583	12,599	$12,508
**F**	V80A3^c^L3^c^EM/ PHEA^d^	$464.261	$553.511	13,182	$100.872	840	$120,028

^a^ Assumes cost of vaccination series was USD 40 and duration of protection was 10 years. All costs were measured in 2013 USD. DALYs were discounted at 3%

^b^ Compared with the preceding non-dominated strategy; small differences due to rounding error

^c^ Discrete applications of limited duration (1 day) at start of and during dengue season over 5 years

^d^ 25% reduction in carrying capacity of immature stages, *K* over 1 year

^e^ No intervention–steady state in Thailand, all ages combined

A3 = adulticide (3 applications); D = dominated; DALY = disability-adjusted life-year; ED = extended dominance; L3 = larvicide (3 applications); USD = United States Dollars; V80 = vaccination with 80% coverage

Accordingly, the expected ICER for Strategy E was the only combined control strategy that could be considered cost-effective under the criteria of 3 × GDP per capita, although not under alternative threshold criteria for cost-effective interventions in Thailand [112].

When considering the payer perspective, similarly, only Strategy E was deemed cost-effective ($13,254 vs. Strategy C–adulticide and EM/ PHEA) under the metric of 3 × GDP per capita.

### Scenario analyses–outcomes

In this section, we broaden our analyses to consider the impact of *Wolbachia* alone and, subsequently, in combination with vaccination. It was assumed that *Wolbachia*-infected mosquitoes have arrived to fixation in the (mosquito) population and that vaccine coverage has arrived at steady state, i.e. there was no ramp-up period.

A decrease of approximately 84% in disease burden (67,595 to 10,623 DALYs lost) was observed compared to the expected burden of dengue disease over 10 years (DALYs lost) in the base-case steady state without interventions. When *Wolbachia* was combined with vaccination (low coverage [40%] scenario–Strategy WV40), medium coverage [60%] scenario–Strategy WV60 or high coverage [80%] scenario–Strategy WV80), only relatively modest incremental reductions in disease burden were predicted (Table 8). As alluded to previously, one potential explanation for this is that vector control, in this case *Wolbachia*, has reduced the force of infection to such an extent that the additional impact of vaccination may only be marginal.

**Table 8:** Impact of *Wolbachia* and combined *Wolbachia* vaccination on dengue burden over a 10-year period (number of cases, outpatient consultations, hospitalisations, deaths and DALYs lost).

Category	*Wolbachia W*	*Wolbachia* / Vaccination (Low–WV40)^a^	*Wolbachia* / Vaccination (Medium–WV60)^a^	*Wolbachia* / Vaccination (High–WV80)^a^
Total dengue cases (millions)	1.123	1.094	1.080	1.066
Symptomatic	1.050	1.023	1.010	0.997
Severe	0.073	0.071	0.070	0.069
Total outpatient consultations (millions)	1.025	0.999	0.986	0.973
Total hospitalisations (millions)	0.098	0.096	0.094	0.093
Total deaths	140	136	134	133
Total DALYs lost	10,623	10,346	10,213	10,081
Total DALYs lost per million population	167	163	161	159

^a^ *Wolbachia* combined with vaccination (40% vaccination coverage, 60% vaccination coverage, 80% vaccination coverage for Strategies WV40, WV60 and WV80 respectively). Vaccination and *Wolbachia* assumed to have arrived at steady state/ fixation.

DALY = disability-adjusted life-year

**Fig 1:**
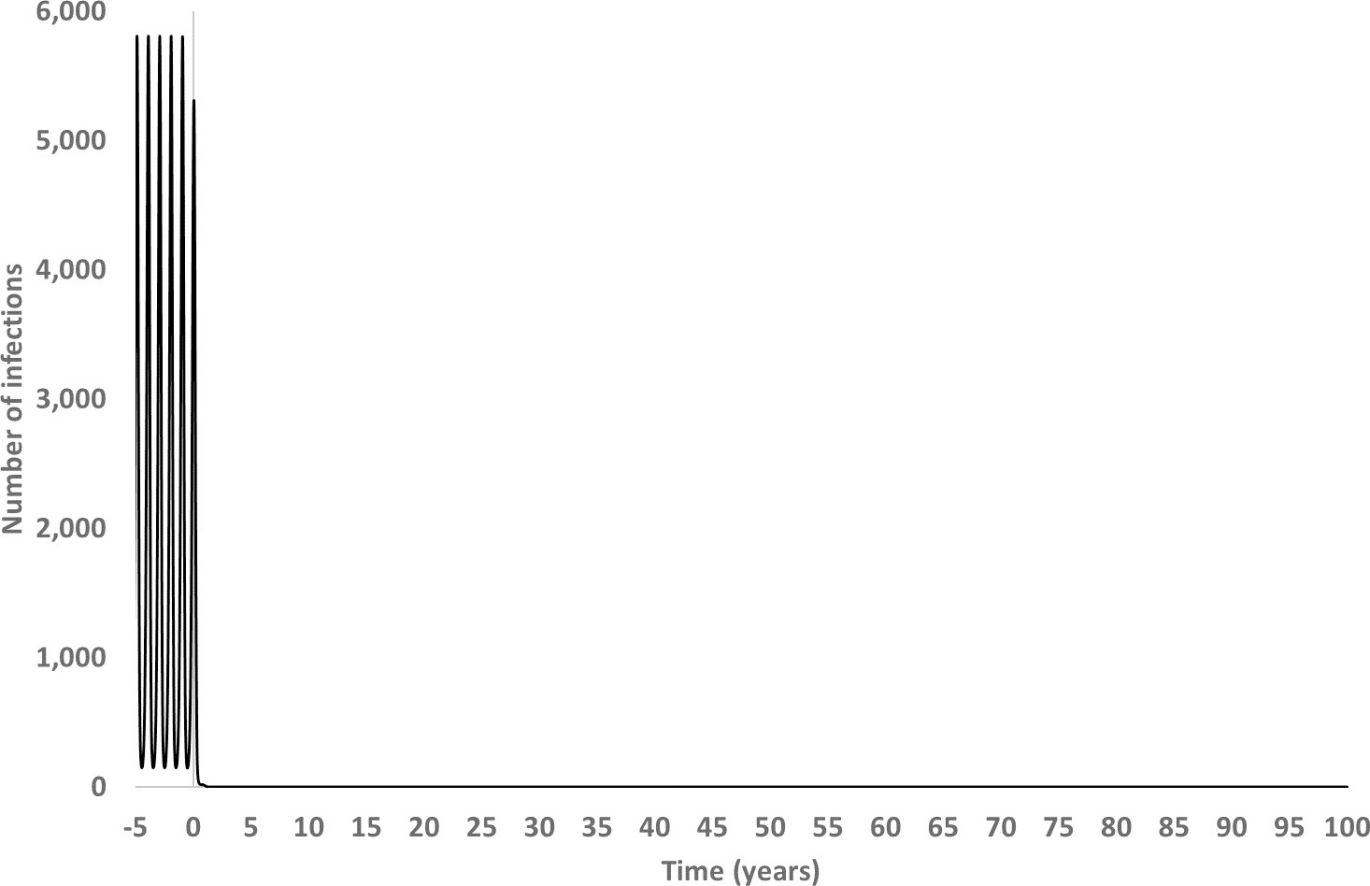
Long term impact of combined *Wolbachia vaccination* on dengue burden (number of cases). *Wolbachia* combined with high-coverage vaccination (80% vaccination coverage). Vaccination and *Wolbachia* assumed to have arrived at steady state/ fixation.

If we considered a longer timeframe and extended the period of follow-up to approximately 100 years, model simulations predicted that with *Wolbachia* alone, dengue disease was suppressed for more than 25 years before any meaningful rebound in incidence was observed. When *Wolbachia* was combined with targeted vaccination (i.e. low coverage vaccination scenario–Strategy WV40), this period was approximately doubled to just over 50 years. Correspondingly, when combined with broader vaccination coverage (e.g. in the range 60–80% of target vaccine population), the period of dengue disease suppression was extended to approximately 100 years (Fig 1).

### Scenario analyses–cost-effectiveness analysis

In this section, we extended cost-effectiveness analyses to also include *Wolbachia* and vaccination combinations. In the first instance, we evaluated the cost-effectiveness of *Wolbachia*, alone and in combination with vaccination using a *Wolbachia* cost of $1 per dengue case averted and subsequently using a *Wolbachia* cost of $1 per person. As mentioned previously, it was assumed that *Wolbachia*-infected mosquitoes had arrived to fixation in the (mosquito) population and that vaccine coverage had arrived at steady state, i.e. there was no ramp-up period.

Initially, we examined the cost-effectiveness of *Wolbachia* singularly compared to the other interventions in our analyses. From the societal perspective, EM/ PHEA formed the reference control as it was the least costly intervention. Total discounted costs (10 years) for *Wolbachia* amounted to approximately $347 million, of which 79% ($274 million) comprised intervention costs. *Wolbachia* was the only non-dominated intervention, with an ICER of $343 per DALY averted; all other singular interventions were dominated (Table 9). When restricting the analysis to the payer perspective (i.e. including only direct medical costs for inpatient and outpatient dengue cases), EM/ PHEA similarly formed the reference intervention with *Wolbachia* being the only non-dominated control with an ICER of $1,399 per DALY averted.

**Table 9:** Cost-effectiveness analysis of single dengue control strategies including *Wolbachia* (societal perspective).^a^

Strategy	Intervention Costs (millions)	Discounted total		Incremental		$/ DALY Averted^b^
		Intervention + Societal Costs (millions)	DALYs Lost	Intervention + Societal Costs (millions)	DALYs Lost	
EM/ PHEA^c^	$24.288	$332.598	51,670			
*Wolbachia*	$273.744	$346.696	10,623	$14.099	41,048	$343
Adulticide^d^ (× 3)	$106.065	$362.673	41,731	-	-	D
No intervention^e^	$0.000	$412.265	67,595	-	-	D
Vaccination (80%)	$273.135	$427.314	23,372	-	-	D
Larvicide^d^ (× 3)	$106.065	$470.216	59,788	-	-	D

^a^ All costs were measured in 2013 USD; costs and DALYs were discounted at 3%

^b^ Compared with the preceding non-dominated strategy; small differences due to rounding error

^c^ 25% reduction in carrying capacity of immature stages, *K* over 1 year

^d^ Discrete applications of limited duration (1 day) at start of and/ or during dengue season over 5 years; 3 applications per dengue season for 5 years

^e^ Steady state

D = dominated; DALY = disability-adjusted life-year; EM/ PHEA = environmental management/ public health education and awareness

Accordingly, our results suggest that from both the payer and societal perspectives, a *Wolbachia* programme (wMel) can be considered a potentially cost-effective (if not highly cost-effective) intervention in the setting of Thailand.

In this section, we present cost-effectiveness results for the simultaneous comparison of multiple dengue control strategies including *Wolbachia* (Table 10).

**Table 10:** Cost-effectiveness analysis of alternative combined dengue control strategies including *Wolbachia* (societal perspective).^a^

Strategy	Interventions	Intervention Costs (millions)	Discounted Total		Incremental		
			Intervention + Societal Costs (millions)	DALYs Lost (thousands)	Intervention + Societal Costs (millions)	DALYs Lost (thousands)	$/ DALY Averted^b^
C	A3^c^EM/ PHEA^d^	$130.353	$295.056	26,621	–	–	–
D	A3^c^L3^c^EM/ PHEA^d^	$236.418	$371.576	21,404	–	–	ED
A	No intervention^e^	$0.000	$412.265	67,595	–	–	D
B	A3^c^L3^c^	$212.130	$424.679	34,223	–	–	D
WV40	WolVacc40^f^	$410.504	$481.592	10,346	$186.536	16,275	$11,462
E	V80A3^c^EM/ PHEA^d^	$403.817	$487.143	12,256	–	–	ED
WV60	WolVacc60^f^	$478.888	$549.071	10,213	$67.478	134	$503,966
F	V80A3^c^L3^c^EM/ PHEA^d^	$509.895	$590.420	11,814	–	–	D
WV80	WolVacc80^f^	$547.272	$616.568	10,081	$67.498	131	$514,432

^a^ Assumes cost of vaccination series was USD 40 and duration of protection was 10 years. All costs were measured in 2013 USD; costs and DALYs were discounted at 3%

^b^ Compared with the preceding non-dominated strategy; small differences due to rounding error

^c^ Discrete applications of limited duration (1 day) at start of and during dengue season over 5 years

^d^ 25% reduction in carrying capacity of immature stages, *K* over 1 year

^e^ No intervention–steady state in Thailand, all ages combined

^f^ *Wolbachia* combined with vaccination (40% vaccination coverage, 60% vaccination coverage, 80% vaccination coverage for Strategies WV40, WV60 and WV80 respectively). Vaccination and *Wolbachia* assumed to have arrived at steady state/ fixation

A3 = adulticide (3 applications); D = dominated; DALY = disability-adjusted life-year; ED = extended dominance; L3 = larvicide (3 applications); V80 = vaccination with 80% coverage

When considering *Wolbachia* combined with vaccination, total (10-year) societal costs were estimated at $482 million, $549 million and $617 million for *Wolbachia and* low (Strategy WV40), medium (Strategy WV60) and high (Strategy WV80) vaccination coverage scenarios, respectively. The main cost drivers were the costs of *Wolbachia* ($274 million) and vaccination ($137 million, $205 million and $274 million for low, medium and high vaccination coverage respectively).

Strategy C (adulticide and EM/ PHEA) was the least costly strategy and therefore acted as the reference. Only *Wolbachia* and vaccination combination strategies were non-dominated, with all other combined control strategies under evaluation (i.e. B, D, E and F) being dominated. The expected ICER for Strategy WV40 was the only control strategy that met wider criteria to be considered potentially cost-effectiveness from both the societal ($11,462 per DALY averted vs. Strategy C) and payer ($12,520 per DALY averted vs. Strategy C) perspectives.

Considering the cost-effectiveness of *Wolbachia*, singularly and in combination with vaccination, using a *Wolbachia* cost of $1 per person, total (10-year) discounted costs from the societal perspective (inclusive of intervention costs) were estimated at approximately $134 million, $333 million, $431 million and $530 million for single *Wolbachia* and combined with low, medium and high coverage vaccination scenarios, respectively. As a single intervention, *Wolbachia* was the most economical option from both the societal and payer perspectives, with all other single dengue control strategies being dominated. In combination with a low coverage vaccination scenario, *Wolbachia* (Strategy WV40) was the least costly strategy from the societal perspective and acted as the reference. All other control strategies were dominated except for Strategies WV60 and WV80.

### Sensitivity analysis

**Fig 2:**
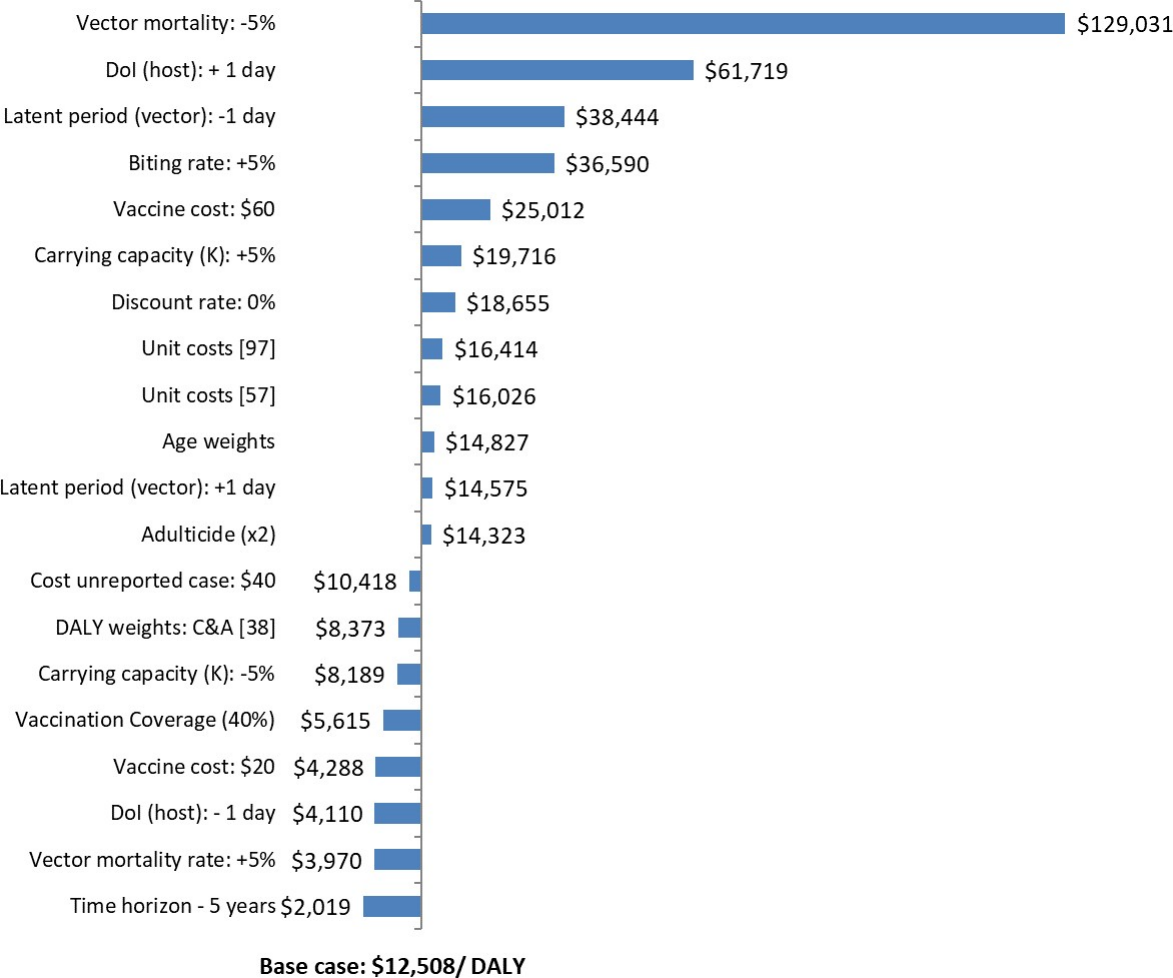
Deterministic sensitivity analysis (societal perspective). DoI = duration of infectiousness (host); DoP = duration of vaccine protection.

Fig 2 summarises the univariate deterministic sensitivity analysis performed on our model. The tornado diagram is shown for Strategy E (vaccination [80% coverage]/ adulticide [3 interventions] and EM/ PHEA) vs. Strategy C (adulticide [3 interventions] and EM/ PHEA]) only as it was not possible to present a tornado diagram for other ICERs in the table of incremental analyses. The model was most sensitive to key epidemiological parameters, particularly vector mortality rate (–5%: $129,031 per DALY averted) and duration of host infectiousness (+1 day: $61,719 per DALY averted) and led to corresponding rises in dengue cases and DALYs lost. The next two most influential parameters were again epidemiological variables, vector latent period (+1 day) and biting rate (+5%) and similarly, also led to rises in dengue cases and DALYs lost. This resulted in analogous increases in the baseline ICER to $38,444 and $36,590 per DALY averted, respectively. All of the latter ICERs were in excess of threshold criteria for cost-effectiveness. With the exception of vaccine cost of $60 ($25,012 per DALY averted), increase (+5%) in carrying capacity (K) ($19,716 per DALY averted) and 0% discount rate ($18,655 per DALY averted), the rest of the parameters under examination (amounting to 24 scenarios in total) and predominantly consisting of economic variables, yielded ICERs within the broader criteria for cost-effectiveness (i.e. 1 ×, 2 × or 3 × GDP per capita). At the lower end of the scale, a number of scenarios led to a reduction in the base-case cost-effectiveness ratio from cost-effective to highly cost-effective (i.e. 1 × GDP per capita) including 5-year time horizon (in that vaccine coverage was still in the ramping-up stage at 5-year follow-up), vector mortality rate (+5%) and vaccine costs of $20 ($4,288).

## Discussion

In this study, we assessed the epidemiological and economic impact of a range of possible dengue control interventions, both singularly and in combination, using a previously developed mathematical model [62]. We used cost-effectiveness analysis to identify the dengue disease control strategies (of the options considered) that have the potential to generate the greatest improvements in disease reduction for the least resources. We focused primarily on historical forms of vector control including adulticide, larvicide and EM/ PHEA before introducing dengue vaccination in the fashion of staggered roll-out over time (consistent with Integrated Dengue Management [60,116,117]). We additionally examined the potential impact and cost-effectiveness of *Wolbachia* as a vector control strategy in exploratory scenario analyses.

The base age-structured epidemiological model was shown to calibrate well at steady state with reported symptomatic and severe dengue cases in different age groups in Thailand from the years 2008–2012 [69] adjusted for under-reporting [70,115]. Additionally, the model predicted outpatient consultations and hospitalisations over 10 years that were consistent with observed data when adjusted for under-reporting. We estimated 1064 DALYs lost per million persons over 10 years (average annual dengue burden of 106 DALYs lost per million persons). This would appear to be on the lower side of other published estimates. For example, Clark et al. [12] estimated a total of 427 DALYs lost per million persons in 2001. The difference is primarily due to both the greater number of cases (124,409) and deaths (209) reported in the 2001 Thailand dengue surveillance data used in their study as well as the inflation factor (10) employed to account for under-reporting.

Our base-case model simulations predicted that single control strategies (adulticide OR vaccination) and a combined strategy in the form of vaccination/ adulticide/ EM/ PHEA would be highly cost-effective and cost-effective control measures, respectively, consistent with guidance (threshold) criteria for cost-effective health interventions. Exploratory scenario analyses also showed *Wolbachia* (in isolation) to be highly cost-effective vs. other single control measures and exhibited marked decreases in dengue burden, enhanced by the addition of vaccination. Whilst the incremental impact of broader vaccination coverage in addition to *Wolbachia* was relatively limited, it considerably influenced both the costs and cost-effectiveness of a combined control strategy according to cost-effectiveness threshold criteria referred to above.

Base-case findings were robust to variations in assumptions in sensitivity analyses under which ICERs (compared with the preceding non-dominated strategy) were iteratively re-calculated for each change in parameterisation. As expected, epidemiological parameters forming the calibrated dynamic transmission model were most sensitive to variation. Notwithstanding this, cost-effectiveness ratios demonstrated remarkable consistency with base-case analyses. Whilst the ICERs were subject to variation in each re-iterative calculation, the conclusions did not manifestly alter after performing extensive sensitivity analyses.

Our study results are broadly consistent with previous research, although methodological differences would perhaps suggest that it is unlikely that the findings of different studies are directly comparable. Methodological differences that have the potential to impact results include, for example, comparators, the specified efficacy or mortality rate for vector control, duration and intensity of vector control interventions (i.e. continuous, monthly etc.), unit costs, vaccine price, perspective and timeframe, amongst others. For example, with respect to efficacy/ mortality rates, Luz et al. [54] employed mortality rates of 30%, 60% and 90% to characterise low, medium and high efficacy insecticide-based vector control, respectively, whilst Fitzpatrick et al. [59] used only high or medium efficacy vector control strategies in their simulations. This contrasts with the low efficacy profiles for (chemical) vector control used in the current study, which would likely impact the ICERs and perhaps explain some of the elements contributing to their higher nature compared with other authors [54,59]. With respect to unit costs, we used dengue-related costs derived from Shepard et al. [6], although they subsequently updated these estimates in 2016 [7]. Whilst there are differences between the two sets of unit costs, it is unlikely that the current results would change markedly, or that any bias would be introduced, given the broad consistency between the estimates (as long as either Shepard et al. [6] OR Shepard et al. [7] costs–not a mixture–were applied to all comparators under evaluation). When substituting unit costs in the current study for those reported (excluding vaccine costs and/ or vector control costs) in Fitzpatrick et al. [59], Lee et al. [100] and Flasche et al. [42] as part of sensitivity and scenario analyses, the broad order of interventions under evaluation remained unchanged from the base case in all three instances. Specifically, using healthcare unit cost estimates from Fitzpatrick et al. [59], the expected baseline ICER of Strategy E (vaccination, adulticide, EM/ PHEA) vs. Strategy C (adulticide, EM/ PHEA) increased from $12,508 to $16,026 per DALY averted. Similarly, using healthcare unit costs from Lee et al. [100], the resulting ICER for Strategy E vs. Strategy C increased to $16,414 per DALY averted while the same ICER (i.e. Strategy E vs. Strategy C) decreased to $11,271 per DALY averted when using healthcare unit costs presented in Flasche et al. [42]. Notwithstanding these differences, the general direction of study results suggests an inherent consistency across study designs and geographies.

As highlighted previously, comparatively few (although increasing) mathematical modelling studies have historically explored the combined effects of assorted interventions and their impact on the epidemiology of dengue transmission as well as cost-effectiveness. A number of reasons suggest a wider consideration. Firstly, dengue efficacy estimates published to date are variable, with remaining areas of uncertainty. Secondly, it could be argued that even if reported efficacies had been very high, i.e. 80–90%, there would still be a case for some form of mixed strategy that incorporates, but does not rely solely on, vaccination. Yellow fever provides an important reference in this regard in that vaccination is the primary tool for the prevention of yellow fever, with the vaccine recognised as being safe and effective in preventing the disease in different age groups with durable protection. Nevertheless, despite this, estimates from different bodies suggest that there are approximately 200,000 cases and 30,000 deaths linked to yellow fever annually [118] with urban outbreaks leading to the international transmission of yellow fever beyond its historical borders. Vector control embracing public education, surveillance, larva and adult mosquito control are advocated as important aspects in the prevention and control of vector-borne diseases including yellow fever [119]. This would suggest that there is a still place for other forms of vector control in addition to vaccination for the control of yellow fever and, by extension, dengue fever, and mathematical modelling studies can aid in these policy debates.

In considering the scope and potential of mixed dengue control strategies, mathematical modelling can be valuable in exploring ‘what-if’ control scenarios. Such analyses have the potential to assist relevant stakeholders in considering the addition of new interventions and/ or changing the implementation of existing ones as well as assist in characterising what could be expected from implementation of combination interventions. Moreover, the inclusion of cost-effectiveness information seeks to address decision-maker and policy-maker needs in lower-income and middle-income countries, which are increasingly focused on developing evidence-based priority-setting frameworks that incorporate value for money criteria [111,120,121].

The outputs from mathematical model simulations, whilst both informative and necessary, are not sufficient for decision-making purposes and should not be the only gauge to provide the basis for recommendations and/ or changes in policy. A range of criteria as part of a wider evidence generation and synthesis framework also influences the choices and determinations in the allocation of scarce healthcare resources. Whilst cost-effectiveness analyses can assist in the assessment of value for money, they must be considered alongside other health system goals. This includes, but is not limited to, for example, affordability and overall budget impact, equity and feasibility as well as considerations of community participation and acceptance amongst others [111,122].

This study is subject to a number of limitations. Firstly, our transmission model did not account for asymptomatic infections, rather focussed on clinically apparent infections. Asymptomatic infections are thought to form an important element of the dengue burden with some 75% of dengue cases being asymptomatic [1,123–125]. Additionally, asymptomatic cases may also play a role in dengue transmission, potentially acting as a pool of infection, although commentators highlight the absence of ‘clear’ data with respect to viremia in inapparent infections as well as the effect of the latter on dengue transmission [125,126]. Notwithstanding this, the focus of this paper is on the economic impact of symptomatic dengue infections and their abeyance. Hence, we do not believe that this omission fundamentally undermines the broad conclusions of our analyses. We did not adjust for, nor take into consideration, any positive externalities of vector control programmes on the burden of disease and costs of illness associated with other vector-borne diseases (e.g. zika virus, chikungunya, malaria, etc.) in Thailand. This omission would most likely under-estimate the cost-effectiveness of vector control combinations in our analyses. The vaccine profile used in this study was informed by real-world overall efficacy data [35,36]. For simplicity, we did not account explicitly for individual serotypes (i.e. DENV-1, DENV-2, DENV-3 and DENV-4) in our model, rather we simulated consecutive dengue infections. In the use of reported efficacy data [35,36], we applied this to a paediatric cohort rather than the age demographic specified in the trial on the assumption that an age-based indication would subsequently be extended to include younger age cohorts and include paediatric vaccination at 1 year of age and under. We ignored any apparent reported imbalances in vaccine immune response between different serotypes and thus any potential negative implications that may follow from this. Moreover, we assumed reported overall vaccine efficacy was constant post-18 months follow-up and did not lessen over time. This could possibly have led to overestimates of the impact of dengue vaccination in the longer term, although it is felt that general conclusions concerning possible enhancements of vector control programmes from simultaneous vaccination strategies (and vice versa) remain unchanged. Our analyses used short-term intervention horizons (1 and 5 years) for traditional vector control measures under evaluation. As a result, this may have induced the so-called ‘divorce effect’ following the introduction and cessation of non-immunising vector control measures [64,127]. In reality, and as highlighted by previous commentators [128], successful vector control programmes would unlikely be terminated as rapidly or abruptly, although as the authors further indicated, it is not inconceivable that a vector control programme could be interrupted, discontinued and/ or substituted (for another programme) for a variety of reasons. This may plausibly include, for example, funding issues, conflict, natural disasters, insecticide resistance or where an intervention were to be judged ineffective. To mitigate the impact of any divorce effect, it is envisaged that such vector control programmes would continue for an indefinite period and/ or until a mixture of more effective and durable control programmes (e.g. *Wolbachia* and/or the use of irradiated mosquitoes, etc.) would displace and in turn substitute for current vector control practices. To minimise the risk and potential for insecticide resistance as a result of longer-term chemical use associated with vector control, it is recommended that insecticide resistance management strategies are also implemented [129]. These strategies may take the form of insecticide rotation (where frequency of rotation is designed to use different insecticides of different modes of action in order that there is not constant exposure to a single chemical), mosaic (which involves the spatial alternation of 2 or more insecticides with different modes of action) and mixture of insecticides (which involves the simultaneous use of 2 or more insecticides with different modes of action). Qualitative research from Surin, Thailand indicates that most providers actually used a single chemical rather than mixed chemicals (which would be in line with integrated vector management [130]), primarily due to resource constraints [131]. Hence, it would be important to ensure that current protocols are practically implemented before introducing any new initiatives. With reference to exploratory analyses of *Wolbachia* as one element of a dengue control strategy, it is acknowledged that many practical hurdles still exist before a widespread *Wolbachia*-based dengue control strategy can be implemented. These include, for example, the optimal choice of *Wolbachia* strain, appropriate surveillance and monitoring of environmental and evolutionary changes, as well as community ‘buy-in’ and acceptance, amongst others [132,133]. The premise that we are examining is not the ‘how’ of implementation, rather what the possible population impact could be once *Wolbachia*-infected mosquitoes have arrived at equilibrium/ steady state fixation. Although coverage in reality is likely to be limited initially, this exploratory scenario analysis gives some insights into the human population impact of a potential *Wolbachia* programme on a large scale countrywide, both separately but also in combination with vaccination. A further limitation relates to the chosen year of unit costs. Specifically, we used costs for the year 2013 and have not updated these to more recent years, which may suggest that our analyses are slightly out of date. However, it is unlikely that any bias was introduced into our comparative analyses, as the same reference year for costs was applied in all analyses. It also enabled us to compare our results with key published dengue analyses that used 2013 unit costs. Further limitations in relation to the epidemiological transmission model can be found in Knerer et al. [62].

Although much research and discussion has focused on the promise of dengue vaccination, it is now broadly accepted, for various reasons, that even after vaccination roll-out, a multi-faceted approach focused on the integration of control strategies may be warranted [24,134]. Chemical and environmental management interventions have formed the basis of efforts to control dengue fever over the last 50 years in spite of acknowledged limitations in terms of effectiveness, mode of delivery, cost, and duration of sustainability [135,136], but may still have an important role to play in the short to medium term. Accordingly, quantitative analyses presented in this paper are intended to contribute to the wider body of research in this area. In this regard, optimal dengue control strategies–identified through cost-effectiveness analyses–may act to facilitate value for money gains and produce health improvements in the most budget conscious way.

This paper has formed the second part of a three-part series examining the broader impacts of mixed control strategies on the epidemiology of dengue fever in Thailand. In the third part of the series, we will move beyond cost-effectiveness analysis to focus on affordability in the context of constrained optimisation. This is because, the former does not directly address the problem that as Sendi and Briggs [137] have indicated, “…‥decision-makers are increasingly constrained by a fixed budget and may not be able to fund new, more expensive interventions, even if they have been shown to represent good value for money”.

## Supporting information

S1 Appendix (Methods–Additional Details). (DOCX)

S2 Appendix (Dengue CEA Decision Tree Results). (XLSB)
