# Supplementary material for: The economic impact and cost-effectiveness of combined vector-control and dengue vaccination strategies in Thailand: results from a dynamic transmission model
Source: PLoS Negl Trop Dis. 2020 Oct 23;14(10):e0008805. doi: 10.1371/journal.pntd.0008805 (PMC7654761; doi:10.1371/journal.pntd.0008805)
Supplement: S1 Appendix — (DOCX) [file pntd.0008805.s001.docx]

**S1 Appendix – The economic impact and cost-effectiveness of combined vector-control and dengue vaccination strategies in Thailand: results from a dynamic transmission model.**

**Gerhart Knerer, Christine S.M. Currie, Sally C. Brailsford**

# Methods

## Epidemiology Model Structure

The previously published model extends work by Bartley et al. [1] and includes the following elements: consecutive infections with all four serotypes, age-structure of the population, seasonality, cross-protection and cross-enhancement and the impact of combined vector-control and vaccination strategies on the transmission of dengue fever [2]. Model compartments comprise those for both human and vector populations. The human population (N_h_) is divided into susceptible to dengue infection, S_h_; exposed but not yet infectious (i.e. incubating the virus), E_h_; infected and infectious humans, I_h_; temporary cross-protection, C_p_; temporary cross-enhancement (C_E_) (i.e. [enhancement](http://www.biology-online.org/dictionary/Enhancement) of [viral](http://www.biology-online.org/dictionary/Viral) infectiousness caused by antibodies that do not neutralise [3-5]) and immune (R) compartments. The final recovery state R imparts permanent immunity to that serotype, but only temporary immunity to other serotypes.

Human hosts can experience a primary infection with one serotype followed by the possibility of subsequent infections with other serotypes. Accordingly, exposed, infectious and immune states are further stratified by the number of infections suffered (i.e. primary, secondary, tertiary etc.) in the form E_h_, E_h2_, E_h3_ and E_h4_. The life cycle of the mosquito is represented in the model by two developmental phases. The aquatic phase comprising egg, larva and pupa stages is denoted by A_v_. The adult stage is divided into three compartments: number of susceptible mosquitoes, S_v_; number of exposed but not yet infectious mosquitoes (i.e. incubating the virus), E_v_ and infected and infectious mosquitoes, I_v_. The total mosquito population is N_v_ (i.e. N_v_ = S_v_ + E_v_ + I_v_). The epidemiological literature and previous modelling studies that were used to inform parameter values in the model, along with further model details and model inputs, can be found in Knerer et al. [2]. The flow diagram of the infection process is presented below.

**S1 Appendix Figure 1: Flow diagram of the infection process**





^Due to space constraints, the following expression (Ih1 + Ih2 + Ih3 + Ih4 + ((Ihe2φhe) + (Ihe3φhe) + (Ihe4φhe))) is signified by I*. Underlying background mortality (μh) is applied to all compartments but not shown on the figure. Only dengue-induced mortality (μD) is displayed.^

## Data, Under-reporting, Expansion Factors and Calibration

Data. Dengue population level epidemiological data from National Epidemiological Surveillance in Thailand [6] for the years 2008–2012 indicate that there was an average of 82,505 reported cases of dengue per year including 43,890, 1,688 and 36,927 dengue haemorrhagic fever, dengue shock syndrome and dengue fever infections, respectively. An average of 88 deaths per year were reported in the period 2008–2012 with the great majority (72%) due to dengue shock syndrome with the remainder attributable to dengue haemorrhagic fever. The highest number of cases was in the 15–24 years age group (n = 21,840) followed by the 10–14 years age group (n = 20,367).

Under-reporting/ Expansion Factors. The issue of under-reporting of dengue cases, akin to missing data, has implications for the development of mathematical models seeking to estimate the burden of disease. Our model seeks to calculate the ‘true’ epidemiological burden of dengue fever in Thailand by incorporating an adjustment for estimated under-reporting. Research indicates potential under-reporting of total cases of symptomatic dengue infections, which are not reflected in national surveillance figures [7,8]. Undurraga et al. [7] documents an average reporting rate of approximately 13% of total symptomatic dengue episodes in South East Asia. This suggests an overall expansion factor (EF) of 7.6 in South East Asia to convert reported cases into estimated ‘actual’ cases, i.e. total dengue symptomatic infections [7]. Expansion factors for individual countries in South East Asia range from approximately 3.8 in Malaysia to 19 for East Timor with a proposed EF for Thailand of 8.5 [7]. An EF of 2.9 is advised for inpatient dengue cases in Thailand, consistent with Wichmann et al. [8] who recommended EFs of 2.6 and 8.7 for inpatient and total dengue cases in Thailand respectively. More recently, Nealon et al. [9] calculated expansion factors for symptomatic dengue disease in individual countries based on the active phase of the CYD14 trial [10] which varied according to case definitions (different laboratory or clinical criteria). For Thailand, these were 12.0, 8.6 and 8.8 for virologically confirmed dengue, clinically and virologically and confirmed dengue, and clinically diagnosed dengue respectively. Consistent with the above, we adjust the average number of reported cases in the period 2008–2012 (82,505 cases per year stratified by age group) by an expansion factor of 8.5 applied to all age groups to derive total ‘actual’ symptomatic dengue cases.

Calibration. Model estimates were calibrated with figures reported by National Epidemiological Surveillance in Thailand in 2008-2012 [6] multiplied by an expansion factor to adjust for under-reporting. The age specific transmission rate provided the calibration target and the log-likelihood was used as the criterion to evaluate the goodness of fit of candidate models. Starting values for the parameters were based on a focused review of the literature and varied within the ranges of the values identified to determine best fit. S1 Appendix Figure 2 presents the results of the model calibration and sensitivity analyses, which indicate a good fit between observed and predicted data by age group. It is possible that a better fit may exist in individual age groups but if one considers the total log-likelihood (calculated by summing across age groups for each candidate model), the chosen model proved to be the best fit. The latter was obtained for a model with cross-protection only and without the inclusion of cross-enhancement. We did test and compare various levels of cross-enhancement ranging from a 2-fold to 5-fold increase in infectiousness to reflect the potential impact of antibody-dependent enhancement [3-5], but none afforded an improved fit compared to the base model with cross-protection only. We estimate the probabilities of death and of being an ambulatory and/ or inpatient case (inpatient versus outpatient), to replicate the number of deaths and type of treatment reported in national data [6].

**S1 Appendix Figure 2: Predicted vs. observed cases: adjusted for under-reporting**


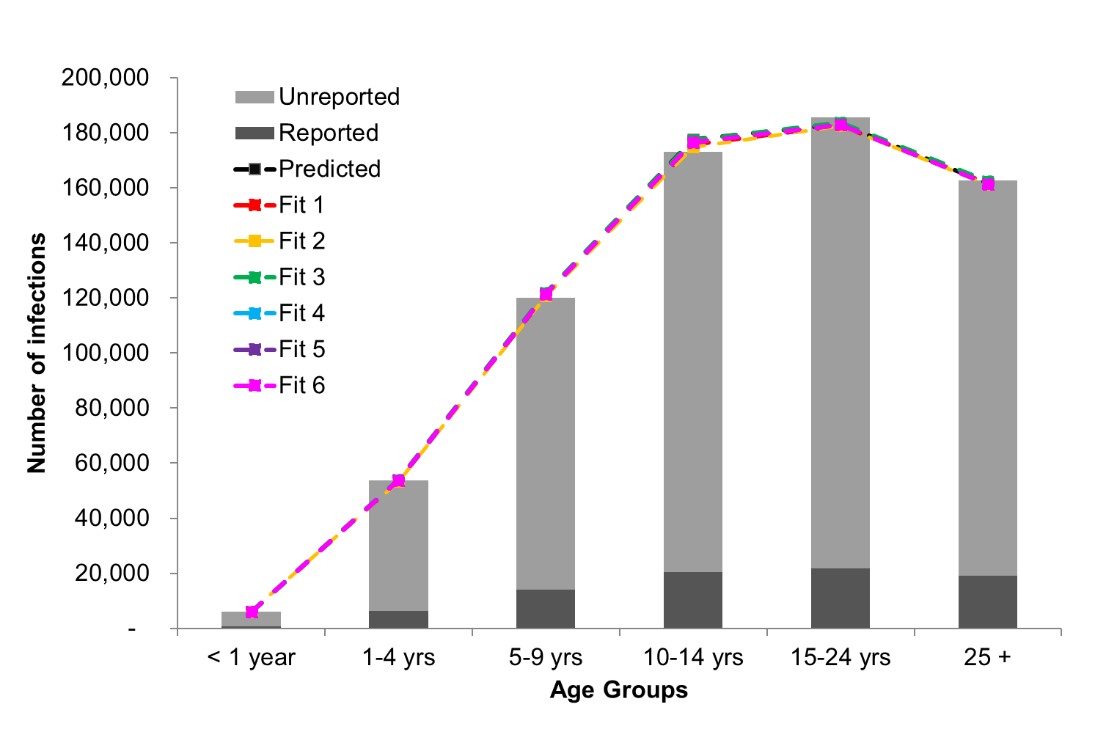


## Outcomes

Disability-adjusted life-years (DALYs) lost to disease were calculated using the methodology described by Murray [11,12].

S1 Appendix Table 1 presents the inputs used to calculate DALYS lost where K is the age weighting modulation factor, D is the disability weight; r is the social discount rate, a is the age at the onset of disability/ average age at death; L is the duration of the disability or the years of life lost due to premature death; C is the age-weighting correction constant and b is the parameter from the age-weighting function, which represents the value of life at different ages. The disability weight reflects the severity of the health state on a scale from 0 (perfect health) to 1 (death). The parameter L represents the duration of the disease (or the years of life lost in the case of dengue death). The remaining years of life were calculated based on the average age of the onset of symptoms in each age group and an average life expectancy of 70 years in Thailand.

Many studies do not incorporate age-weighting as the concept of age-weighted DALYs is often considered controversial. The case where there is no age weighting correspond to the situation where K=0 (no age weights).

**S1 Appendix Table 2: Inputs used to calculate DALYs lost due to dengue in Thailand (2008–2012)**

| Parameter | Value | Source |
| --- | --- | --- |
|  |  |  |
| Age-weighting modulation factor *K* | 1 | [11] |
| Age-corrected constant *C* | 0.16243 | [12] |
| Age-weighting parameter *b* | 0.04 | [12] |
| Disability weight (death) *D* | 1 | [12] |
| Social discount rate *r_O_* | 0.03 | [12] |
| Cost discount rate r*_C_* | 0.03 | [13,14] |
| Duration of disability in reported DF cases (days) *L_DF_* | 10 | [15] |
| Duration of disability in reported DHF/ DSS cases (days) *L_DHF_* | 14 | [15] |
| Duration of disability in unreported cases (days) *L_DFU_* | 4 | [15,16] |
| Years of life lost in death (0–11 months) | 69.5 | Assumption |
| Years of life lost in death (1–4 years) | 67 | Assumption |
| Years of life lost in death (5–9 years) | 63 | Assumption |
| Years of life lost in death (10–14 years) | 58 | Assumption |
| Years of life lost in death (15–24 years) | 50 | Assumption |
| Years of life lost in death (25 + years) | 22 | Assumption |
| Age at onset of disability (0–11 months) | 0.5 | Assumption |
| Age at onset (1–4 years) | 3 | Assumption |
| Age at onset (5–9 years) | 7 | Assumption |
| Age at onset (10–14 years) | 12 | Assumption |
| Age at onset (15–24 years) | 20 | Assumption |
| Age at onset (25 + years) | 48 | Assumption |
|  |  |  |

# References

**1.** Bartley LM, Donnelly CA, Garnett GP. The seasonal pattern of dengue in endemic areas: mathematical models of mechanisms. Trans R Soc Trop Med Hyg. 2002;96: 387-397.

**2.** Knerer G, Currie CS, Brailsford SC. Impact of combined vector-control and vaccination strategies on transmission dynamics of dengue fever: a model-based analysis. Health Care Manag Sci. 2015;18: 205-217.

**3.** Halstead SB. Dengue. Lancet. 2007;370: 1644-1652.

**4.** Aguiar M, Stollenwerk N, Kooi BW. Torus bifurcations, isolas and chaotic attractors in a simple dengue fever model with ADE and temporary cross immunity. Int J Computer Mathematics. 2009;86: 1867-1877.

**5.** Aguiar M, Ballesteros S, Kooi BW, Stollenwerk N. The role of seasonality and import in a minimalistic multi-strain dengue model capturing differences between primary and secondary infections: complex dynamics and its implications for data analysis. J Theor Biol. 2011;289: 181-196.

**6.** Bureau of Epidemiology. Annual Reports for 2008-2012. Available from: <http://203.157.15.110/boeeng/annual.php>

**7.** Undurraga EA, Halasa YA, Shepard DS. Use of expansion factors to estimate the burden of dengue in Southeast Asia: a systematic analysis. PLoS Negl Trop Dis. 2013;7: e2056.

**8.** Wichmann O, Yoon IK, Vong S, Limkittikul K, Gibbons RV, Mammen MP, et al. Dengue in Thailand and Cambodia: an assessment of the degree of underrecognized disease burden based on reported cases. PLoS Negl Trop Dis. 2011;5: e996.

**9.** Nealon J, Taurel AF, Capeding MR, Tran NH, Hadinegoro SR, Chotpitayasunondh T, et al. Symptomatic Dengue Disease in Five Southeast Asian Countries: Epidemiological Evidence from a Dengue Vaccine Trial. PLoS Negl Trop Dis. 2016;10: e0004918.

**10.** Capeding MR, Tran NH, Hadinegoro SR, Ismail HI, Chotpitayasunondh T, Chua MN, et al. Clinical efficacy and safety of a novel tetravalent dengue vaccine in healthy children in Asia: a phase 3, randomised, observer-masked, placebo-controlled trial. Lancet. 2014;384: 1358-1365.

**11.** Murray CJ. Quantifying the burden of disease: the technical basis for disability-adjusted life years. Bull World Health Organ. 1994;72: 429-445.

**12.** Murray CJ, Lopez AD. The incremental effect of age-weighting on YLLs, YLDs, and DALYs: a response. Bull World Health Organ. 1996;74: 445-446.

**13.** Chaikledkaew U, Teerawattananon Y, Kongpittayachai S, Suksomboon N. Thailand’s National Health Technology Assessment Guidelines. 1st ed. Nonthaburi: The Graphico Systems; 2009.

**14.** Edejer TT-T, Baltussen R, Adam T, Hutubessy R, Acharya A, Evans DB, et al. Making choices in health: WHO guide to cost-effectiveness analysis. Available from: <http://www.who.int/choice/publications/p_2003_generalised_cea.pdf>

**15.** Carrasco LR, Lee LK, Lee VJ, Ooi EE, Shepard DS, Thein TL, et al. Economic impact of dengue illness and the cost-effectiveness of future vaccination programs in Singapore. PLoS Negl Trop Dis. 2011;5: e1426.

**16.** Clark DV, Mammen MP, Jr., Nisalak A, Puthimethee V, Endy TP. Economic impact of dengue fever/dengue hemorrhagic fever in Thailand at the family and population levels. Am J Trop Med Hyg. 2005;72: 786-791.
